# Ultrasensitive MoS_2_ photodetector by serial nano-bridge multi-heterojunction

**DOI:** 10.1038/s41467-019-12592-w

**Published:** 2019-10-16

**Authors:** Ki Seok Kim, You Jin Ji, Ki Hyun Kim, Seunghyuk Choi, Dong-Ho Kang, Keun Heo, Seongjae Cho, Soonmin Yim, Sungjoo Lee, Jin-Hong Park, Yeon Sik Jung, Geun Young Yeom

**Affiliations:** 1 School of Advanced Materials Science and Engineering, Sungkyunkwan University, 2066 Seobu-ro, Jangan-gu, Suwon-si, Gyeonggi-do, 16419 Republic of Korea; 2 SKKU Advanced Institute of Nano Technology (SAINT), Sungkyunkwan University, 2066 Seobu-ro, Jangan-gu, Suwon-si, Gyeonggi-do, 16419 Republic of Korea; 3 School of Electrical and Electronic Engineering, Nanyang Technological University, 50 Nanyang Avenue, 639798 Singapore, Singapore; 4 School of Electronic and Electrical Engineering Sungkyunkwan University, 2066 Seobu-ro, Jangan-gu, Suwon-si, Gyeonggi-do, 16419 Republic of Korea; 5 Department of Electronics Engineering, Gachon University, Gyeonggi-do, 13120 Republic of Korea; 6 Pritzker School of Molecular Engineering, The University of Chicago, 5640 South Ellis Avenue, Chicago, IL 60637 United States; 7 School of Materials Science and Engineering, Korea Advanced Institute of Science and Technology (KAIST), 291 Daehak-ro, Yuseong-gu, Daejeon 305-701 Republic of Korea

**Keywords:** Two-dimensional materials, Nanophotonics and plasmonics

## Abstract

The recent reports of various photodetectors based on molybdenum disulfide (MoS_2_) field effect transistors showed that it was difficult to obtain optoelectronic performances in the broad detection range [visible–infrared (IR)] applicable to various fields. Here, by forming a mono-/multi-layer nano-bridge multi-heterojunction structure (more than > 300 junctions with 25 nm intervals) through the selective layer control of multi-layer MoS_2_, a photodetector with ultrasensitive optoelectronic performances in a broad spectral range (photoresponsivity of 2.67 × 10^6^ A/W at *λ* = 520 nm and 1.65 × 10^4^ A/W at *λ* = 1064 nm) superior to the previously reported MoS_2_-based photodetectors could be successfully fabricated. The nano-bridge multi-heterojunction is believed to be an important device technology that can be applied to broadband light sensing, highly sensitive fluorescence imaging, ultrasensitive biomedical diagnostics, and ultrafast optoelectronic integrated circuits through the formation of a nanoscale serial multi-heterojunction, just by adding a selective layer control process.

## Abstract (web summary)

Fabrication of photodetector devices by selective etching of 2D materials can enable broadband detection. Here, the authors design mono- and multi-layer nano-bridge multi-heterojunction photodetectors based on MoS_2_ with high responsivities of 2.67 × 10^6^ A/W and 1.65 × 10^4^ A/W in the visible–infrared wavelength range and fast photoresponse.

## Introduction

MoS_2_, which is one of the most representative transition metal dichalcogenide (TMD) materials, exhibits a band-gap change from 1.29 to 1.9 eV and a transition from an indirect-gap to a direct-gap as the number of layers is decreased from bulk to mono-layer^1^, thereby, various studies on electronic and optoelectronic applications using these characteristics are being carried out^2–6^. Especially, photoexcitation in the mono-layer MoS_2_ with direct-gap properties enables high absorption coefficient and highly effective electron–hole pair generation^7,8^. In addition, MoS_2_ has a lower dark current and a low-noise equivalent power than commercial advanced silicon avalanche photodiodes due to its high band-gap and high electrostatic control at the atomic-scale thickness, which is very good for low-light detection^9,10^. Therefore, it can be applied to next generation optoelectronic devices in a wide range of fields.

Since the report of a single-layer MoS_2_ photodetector^11^, recently, studies on MoS_2_-based photodetectors with various structures/functionalization such as mechanically exfoliated MoS_2_^7,12^, gate functionalization^13–15^, high-κ (Al_2_O_3_ and HfO_2_) encapsulation^16,17^, phase transition (2H-1T)^18,19^, p-type (AuCl_3_)^20^ or n-type [PPh_3_ and (3-aminopropyl) triethoxysilane (APTES)]^21,22^ doping, perovskite heterostructure^10,23^, hybrid structure consisting of MoS_2_/PbS^24^, MoS_2_/TiO_2_/PbS^25^, MoS_2_/TiO_2_/HgTe^26^ quantum dots and MoS_2_/Rhodamine 6 G (R6G)^27^, and van der Waals (vdW) heterojunction consisting of MoS_2_/black phosphorus (BP)^28^, MoS_2_/graphene^29^, MoS_2_/SnSe^30^, MoS_2_/GaTe^31^, and MoS_2_/GaSe^32^ have been actively performed. The photoresponsivity and photoresponse of MoS_2_-based photodetectors have been greatly improved by these various studies. However, both were not compatible at the same time, and photodetection in the IR region has not been successfully achieved so far.

Here, we demonstrate that the MoS_2_ photodetectors with serial nano-bridge multi-heterojunctions, which were fabricated by a selective layer control of multi-layer MoS_2_ without using hybrid structures or doping with other materials, exhibit ultrahigh-performances (high photoresponsivity and fast photoresponse) in the broad detection range [visible–near-infrared (NIR)]. It is also found that the optoelectronic performances of the MoS_2_ multi-heterojunction devices are improved with the increase in the number of heterojunctions with the mono-/multi-layer structure while decreasing the junction width, and therefore, by decreasing the heterojunctions to nano-bridge junctions (from 1 to >300 heterojunctions with the junction width from 10 µm to 50 nm). The mechanism on ultrahigh optoelectronic performance of the serial nano-bridge multi-heterojunction photodetector could be identified through the analysis of interlayer gap, work function, and depletion region in the mono-/multi-layer heterojunction.

## Results

### MoS_2_ heterojunction by the selective ALE

**Fig. 1:**
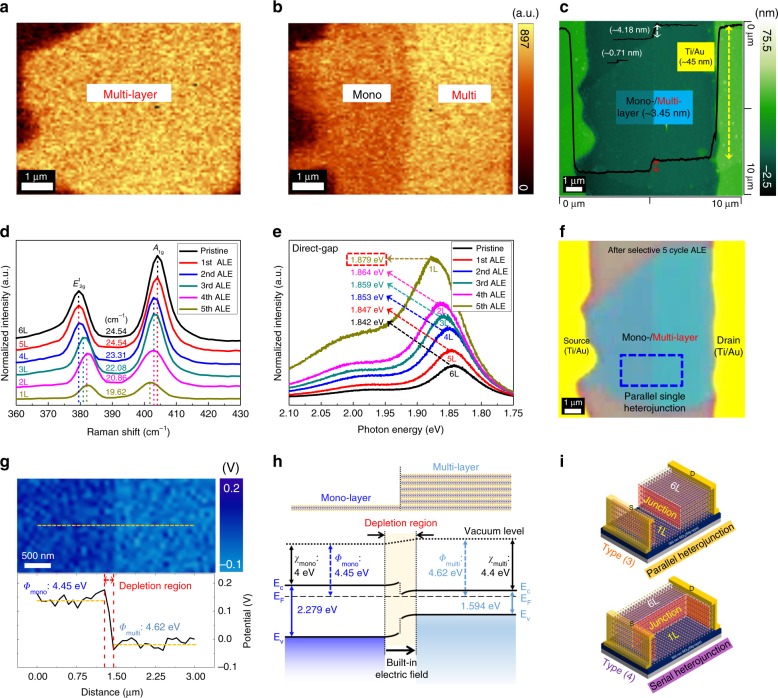
Characterization of heterojunction MoS_2_ formed by the ALE technique. Raman mapping images of multi-layer MoS_2_ **a** pristine 6L-MoS_2_ and **b** the mono-/multi-layer (6 L) MoS_2_ heterojunction after selective 5 cycles of atomic layer etching (ALE) for the left side of the 6L-MoS_2_. **c** Atomic force microscopy (AFM) thickness analysis for the heterojunction in **b**. **d** Raman and **e** photoluminescence (PL) spectra analysis for 1 to 5 cycles of ALE on pristine 6L-MoS_2_. **f** Optical microscopic (OM) image of the mono-/multi-layer heterojunction device. **g** Kelvin probe force microscopy (KPFM) analysis of the mono-/multi-layer heterojunction area (blue box) of the OM image (as shown in **f**). **h** Band diagram for the mono-/multi-layer (6L) heterojunction. **i** The schematic drawing of the MoS_2_ field effect transistors (FETs) for parallel heterojunction [Type (3)] and serial heterojunction [Type (4)]

Before the formation of serial nano-bridge MoS_2_ multi-heterojunctions, we fabricated MoS_2_ single heterojunctions composed of mono-/multi-layer (6L) MoS_2_ by patterning the multi-layer MoS_2_ channel of a multi-layer MoS_2_ back-gated FET with a photoresist (PR) and by selective layer control of the exposed multi-layer MoS_2_ to monolayer. Figure 1a, b show the Raman mapping images of pristine exfoliated multi-layer (6 L) MoS_2_ and the mono-/multi-layer heterojunction MoS_2_ formed by patterning and selective layer controlling of the multi-layer MoS_2_, respectively. The selective layer control of MoS_2_ layers for the formation of mono-/multi-layer MoS_2_ heterojunction was performed by removing the exposed MoS_2_ layers layer-by-layer through atomic layer etching (ALE)^33^ technology (ALE: a cyclic etch method composed of 1st step: Cl radical adsorption and 2nd step: Ar^+^-ion beam desorption per cycle to remove one mono-layer MoS_2_ per each cycle uniformly without damage and contamination. Therefore, to remove 5 MoS_2_ layers from 6-layer MoS_2_, 5 ALE cycles were performed). The schematic diagram of the ALE processing can be seen in Supplementary Note 1. Figure 1c is the atomic force microscopy (AFM) of Fig. 1b and, as shown in the figure, the left side of the MoS_2_ showed the thickness of ~0.71 nm (1L-MoS_2_) while the right side of the MoS_2_ showed the ~4.18 nm (6L-MoS_2_). Also, at the center, the difference between the etched MoS_2_ and unetched multi-layer MoS_2_ was ~3.45 nm indicating the formation of mono-/multi-layer (6L) MoS_2_ heterojunction by the selective layer control of the ALE method. The thickness of the source/drain area deposited with Ti/Au was measured to be ~45 nm. For each cycle of MoS_2_ ALE, using the Raman spectroscopy and photoluminescence (PL) spectroscopy, the change of the MoS_2_ layers per each ALE cycle was measured.

Figure 1d shows Raman spectra of the left MoS_2_ PR open area of Fig. 1b after each ALE cycle, and the change of gap distance between E^1^_2g_ peak and A_1g_ peak from 24.54 cm^−1^ to 19.62 cm^−1^ which indicates the change from 6L to 1L by 5 cycles of ALE can be also seen. The relationship of the gap distance between E^1^_2g_ peak and A_1g_ peak to the MoS_2_ layers has been previously investigated by many researchers^34,35^. In addition, when possible damage, oxidation, and the change of S/Mo ratio on the etched MoS_2_ layer surface by the MoS_2_ ALE were investigated by X-ray photoelectron spectroscopy (XPS), the formation of stable stoichiometric undamaged MoS_2_ layer surface could be observed after the 5 cycles of MoS_2_ ALE (Supplementary Note 2). Figure 1e shows the PL spectra of the left MoS_2_ area of Fig. 1b measured also for each ALE cycle for direct-gap energy measurement, and, with the increase of ALE cycle, the gradual increase of PL intensity and direct-gap energy was observed. Especially, after the 5th ALE cycle, the highest PL intensity and the direct-gap energy of 1.879 eV related to the mono-layer MoS_2_ could be observed^1^. When the PL intensities from the pristine right MoS_2_ area (6L-MoS_2_) and the mono-layer left MoS_2_ area (1L-MoS_2_) of Fig. 1b were measured for the indirect-gap energy measurement (Supplementary Note 3), the PL intensity related to the indirect-gap energy of 1.394 eV was measured for the pristine multi-layer MoS_2_ (6 L) while no PL intensity was observed for the left MoS_2_ area indicating true mono-layer MoS_2_ with a direct-gap. In addition, when the PL analysis results and FET performances of the mono-layer MoS_2_ prepared with 6L-MoS_2_ after 5 ALE cycles are compared with those with mechanically exfoliated pristine mono-layer MoS_2_, almost identical performances have been confirmed within a small error range. Scanning transmission electron microscopy (STEM) for the atomic structure of the mono-layer MoS_2_ fabricated by the ALE process also showed no damage on the mono-layer MoS_2_ such as sulfur vacancy after the ALE process (Supplementary Note 3). Therefore, through the ALE process, the formation of the mono-/multi-layer heterojunction with the staggered alignment having an interlayer gap (narrow gap) has been explicitly identified^36^.

Figure 1f shows optical microscopic (OM) image of the MoS_2_ FET with the mono-/multi-layer heterojunction after the 5 ALE cycles. The variation of the work function in the small rectangular blue box area with the mono-/multi-layer heterojunction of Fig. 1f was measured in Fig. 1g using the Kelvin probe force microscopy (KPFM), and the change of work function from 4.45 to 4.62 eV with a depletion region at the mono-/multi-layer MoS_2_ heterojunction could be identified. The study of a depletion region on a lateral heterojunction using the KPFM has been well investigated by a previous research^37^. Figure 1h shows the schematic band diagram of the fabricated mono-/multi-layer (6L) heterojunction obtained from the differences in work function^38^, electron affinity^38,39^, and electronic band-gap^40^. Due to the built-in electric field in the depletion region of the heterojunction, when a light is irradiated, the photogenerated holes in the mono-layer and multi-layer MoS_2_ go to the multi-layer MoS_2_ region while the photogenerated electrons in the multi-layer MoS_2_ are trapped at the interface and the photogenerated electrons in the mono-layer MoS_2_ stay in the same region away from the interface. Therefore, more photocarriers are expected in the MoS_2_ channel by the enhanced generation of electron–hole pairs compared with the case of channel without the heterojunction. Figure 1i shows the schematic drawing of two different mono-/multi-layer MoS_2_ heterojunctions that can be formed on 6L-MoS_2_ FETs by PR patterning parallel/vertical to the source/drain edges and removing 5L-MoS_2_ selectively as parallel- and serial-type heterojunctions named as Type (3) and Type (4), respectively. [Type (1) is the MoS_2_ FET with a mono-layer MoS_2_ channel and Type (2) is the FET with a multi-layer (6L) MoS_2_ channel].

### Parallel- and serial-type nano-bridge multi-heterojunction by the selective ALE

**Fig. 2:**
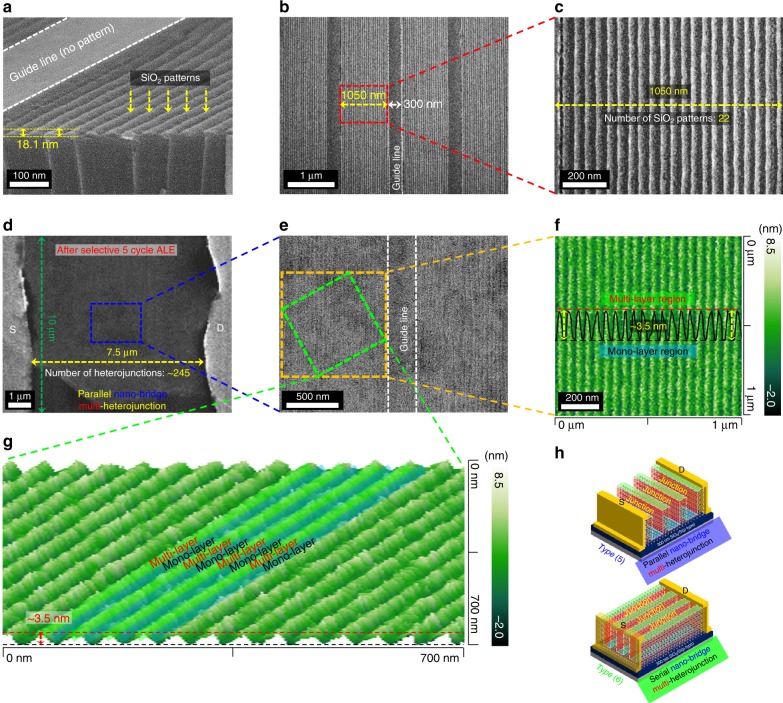
Structural morphology analysis of MoS_2_ FETs with parallel and serial nano-bridge. **a** Side-view and **b** top-view field-emission-scanning electron microscope (FE-SEM) images of 25 nm (50 nm pitch) SiO_2_ line patterns fabricated by a nano-patterning process on multi-layer MoS_2_ FETs. **c** High magnification FE-SEM image for the red box area in **b**. **d** FE-SEM image (after the removal of SiO_2_ mask) of multi-heterojunctions after the selective 5 ALE cycles on the multi-layer MoS_2_ FET patterned with 50 nm pitch SiO_2_ line nano-patterns. **e** High magnification FE-SEM image for the blue box area in **d**. **f** AFM thickness analysis of the multiple MoS_2_ mono-/multi-layer heterojunction for the orange box area in **e**. **g** 3D AFM image for the green box area in **e**. **h** The schematic drawing of MoS_2_ FETs fabricated with parallel nano-bridge multi-heterojunction [Type (5)] and serial nano-bridge multi-heterojunction [Type (6)]

To study the effect of different MoS_2_-based heterojunctions on the optoelectronic performances of back-gated MoS_2_ FETs (photodetectors), in addition to the above parallel- and serial-heterojunctions named as Type (3) and Type (4), parallel nano-bridge type [Type (5)] and serial nano-bridge type [Type (6)] mono-/multi-layer heterojunctions were formed through the selective ALE after patterning the multi-layer MoS_2_ with a nano-patterning process [using a solvent-assisted nanotransfer printing (S-nTP) process]^41^. Figure 2a shows the side-view field emission-scanning electron microscopy (FE-SEM) image of SiO_2_ patterns formed on the 6L-MoS_2_ by the nano-patterning process. 18.1 nm thick SiO_2_ line patterns with 50 nm intervals and the line width of 25 nm could be seen. (In the S-nTP process, for the linear alignment of SiO_2_ line nano-patterns, a guideline shown as white dots which was not patterned with the SiO_2_ nano-patterns was required). Figure 2b, c are the top-view FE-SEM images of SiO_2_ line nano-patterns showing that the width of the SiO_2_ pattern area is 1050 nm having 22 SiO_2_ line patterns with 50 nm pitch and the width of the guideline is 300 nm. Therefore, SiO_2_ line pattern area and the guideline were also repeated with the pitch of 1350 nm.

The SiO_2_ nano-patterns were formed on the multi-layer MoS_2_ FETs parallel or vertical to the source/drain and Fig. 2d, e show the top-view FE-SEM images of the parallel mono-/multi-layer multi-heterojunction (that is, parallel nano-bridges) formed on 6L-MoS_2_ FET after the selective 5 ALE cycles followed by the removal of SiO_2_ masks using a HF solution (HF:deionized water = 1:100, etch time = 10 s). The channel length between the source and drain was ~7.5 µm for the parallel nano-bridges, therefore, the number of 50 nm nano-bridges (one nano-bridge is consisted of 25 nm wide mono-/25 nm wide multi-layer heterojunction) was ~245. Figure 2f, g show the AFM data on the orange box area and green box area in Fig. 2e, respectively, and the thickness differences between the top and the bottom of nano-bridges were uniformly ~3.5 nm indicating the formation of 1L/6L MoS_2_ heterojunctions. For the serial mono-/multi-layer multi-heterojunctions (that is, serial nano-bridges) formed on 6L-MoS_2_ FET by patterning SiO_2_ nano-patterns vertical to the source/drain, the number of the nano-bridges was ~326 because the channel width of the source and drain was ~10 µm. Figure 2h shows the schematic drawing of two mono-/multi-layer MoS_2_ heterojunctions [Type (5)-parallel nano-bridge multi-heterojunction and Type (6)-serial nano-bridge multi-heterojunction] that were formed on 6L-MoS_2_ FETs parallel/vertical to the source/drain edges. Schematic diagram and more details of the fabrication process for the MoS_2_ FET structures of Type (1)–(6) can be seen in Supplementary Note 4.

### Optoelectronic performence of Type (1)–(6)

**Fig. 3:**
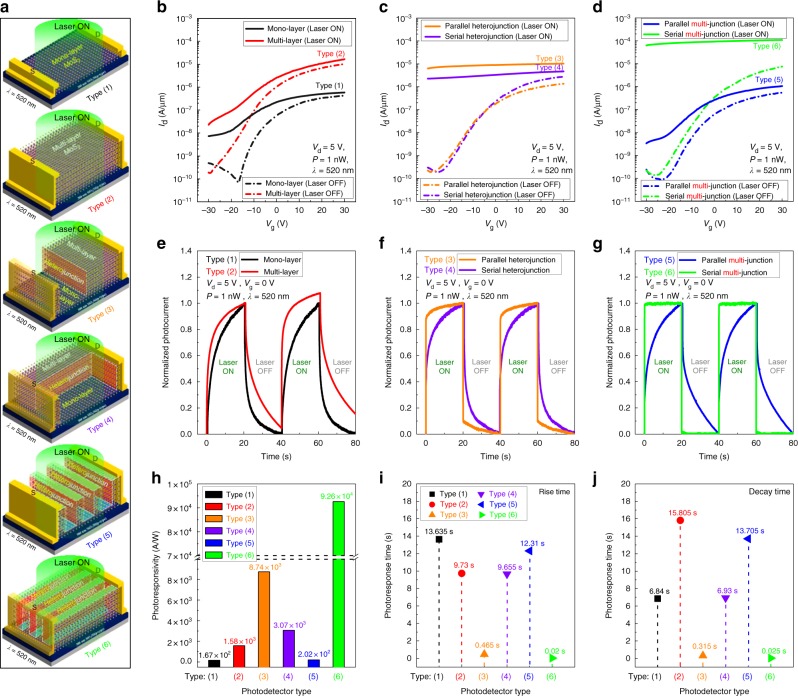
Optoelectronic performance for FETs of Type (1)–(6). **a** Schematic diagram of Type (1)–(6) FETs (photodetectors) irradiated with a laser (*λ* = 520 nm). *I*_d_–*V*_g_ characteristics according to the laser on/off in **b** Type (1), (2), **c** Type (3), (4), and **d** Type (5), (6). The normalized photoresponse curve according to the laser on/off in **e** Type (1), (2), **f** Type (3), (4), and **g** Type (5), (6). **h** The photoresponsivity of Type (1)–(6). **i** Rise time and **j** decay time for the photoresponse of Type (1)–(6)

Using the FET devices fabricated, the optoelectronic properties of the devices were investigated. Figure 3a shows the schematic drawing of the photodetectors with laser irradiation for Type (1)-mono-layer, Type (2)-multi-layer, Type (3)-parallel heterojunction, Type (4)-serial heterojunction, Type (5)-parallel nano-bridge multi-heterojunction, and Type (6)-serial nano-bridge multi-heterojunction. Their optoelectronic properties such as photoresponsivity and photoresponse time are shown in Fig. 3b–g while biasing the device (*V*_d_ = 5 V, *V*_g_ = −30 ~ + 30 V or *V*_g_ = 0 V for the comparison) and irradiating a laser (*λ* = 520 nm, *P*_laser_ = 1 nW). For the photoresponse time, the peak values were normalized to 1.0 for easier comparison and the rise time/decay time of the photoresponse were calculated as shown in Supplementary Note 5. The photoresponse time of Type (1)–(5) excluding Type (6) are relative values for comparison with Type (6) since the steady state was not reached at the laser-on time of 0–20 s. Figure 3b, e show the *I*_d_–*V*_g_ curve and the photoresponse curve, respectively, for the Type (1) mono-layer MoS_2_ FET and Type (2) multi-layer MoS_2_ FET with the laser on/off. As shown in Fig. 3b, the photoresponsivity of Type (2) [1.58 × 10^3^ A/W] was ~9.46 times higher than that of Type (1) [1.67 × 10^2^ A/W] due to the generation of more photocarriers caused by more layers and lower exciton binding energy of the 6L-MoS_2_ (~0.2 eV) compared to 1L-MoS_2_ (~0.4 eV)^42^. The photoresponsivities of the pristine mono-layer and multi-layer MoS_2_ were similar to those investigated by previous researchers^7,21^. In the case of photoresponse curve in Fig. 3e, the rise time during the laser-on time of 0–20 s was shorter for Type (2) [9.73 s] compared to Type (1) [13.635 s] also due to the generation of more photocarriers for the multi-layer MoS_2_. However, for the laser-off time of 20–40 s, due to the more remaining photocarriers (electrons and holes) not recombined by charge traps^43,44^ and the slower electron–hole recombination for the indirect energy band-gap compared to the direct energy band-gap, the decay time was longer for the Type (2) [15.805 s] with the multi-layer MoS_2_ compared to that of Type (1) [6.84 s].

Figure 3c, f show the *I*_d_–*V*_g_ curve and the photoresponse curve, respectively, for the Type (3)-parallel heterojunction MoS_2_ FET and Type (4)-serial heterojunction MoS_2_ FET with the laser on/off. The photoresponsivities of Type (3) [8.74 × 10^3^ A/W] and Type (4) [3.07 × 10^3^ A/W] in Fig. 3c were ~5.53 and ~1.94 times higher than that of Type (2) [1.58 × 10^3^ A/W], respectively, due to the easier electron–hole pair generation caused by the built-in electric field in the depletion region of the heterojunction, differences in work function and electron affinity, and interlayer gap as shown in Fig. 1h. The use of heterojunctions improved not only the photoresponsivity but also the photoresponse speed. As shown in Fig. 3f, the (rise time/decay time) of Type (3) [0.465/0.315 s] and Type (4) [9.655/6.93 s] were lower (~20.9/~50.2 times) and (~1/~2.3 times), respectively, compared to those of Type (2) [9.73/15.805 s]. The differences between Type (3) and Type (4) are the direction of the heterojunction as parallel and serial to source/drain. And, due to the easier carrier transport for the parallel heterojunction compared to the serial heterojunction, which was caused by the same direction of the source/drain electric field and the built-in electric field for the parallel heterojunction, Type (3)-parallel heterojunction MoS_2_ FET showed faster photoresponse compared to Type (4)-serial heterojunction MoS_2_ FET. Previous studies on parallel heterojunction (mono-/multi-layer heterojunction MoS_2_ flakes) MoS_2_ FETs fabricated on laser thinning also showed the increase of photoresponsivity and decrease of photoresponse (rise/decay times) at the same time^36,45^.

On the contrary, in the case of Type (5)-parallel nano-bridge multi-heterojunction MoS_2_ FET and Type (6)-serial nano-bridge multi-heterojunction MoS_2_ FET, as shown in Fig. 3d, g, extremely high photoresponse characteristics were observed for Type (6) compared to Type (5). As shown in Fig. 3d, compared to Type (2)-multi-layer MoS_2_ FET, the photoresponsivity of Type (6) [9.26 × 10^4^ A/W] was increased ~58.6 times while that of Type (5) [2.02 × 10^2^ A/W] decreased ~7.8 times. The decrease in photoresponse for Type (5) is believed to be related to the ~245 mono-/multi-layer heterojunctions located to parallel to the source/drain, and which acted as series energy barriers and channel scattering source for the carrier transport. The degradation of Type (5) can be identified by the comparison of the electrical characteristics of MoS_2_ FETs calculated by *I*_d_–*V*_g_ characteristics at the laser-off state such as on-current, on/off ratio, and field-effect mobility (*μ*_FE_) among the Type (1)–(6) FETs (Supplementary Note 6). On the contrary, the photodetector of Type (6) has the drift mobility 10 times higher than that of Type (5). The only difference between those two types of devices is found to the direction in constructing the heterojunction. Since there is no energy barrier as in case of Type (5) device, in the carrier transport between source and drain junctions, the electrical characteristics are mostly like those of Type (2), as can be confirmed by Supplementary Note 6. (Previous reports show that the high mobility of the channel material contributes to the fast carrier transport^43,46,47^.) In addition, the significant improvement of photoresponsivity for the MoS_2_ FET of Type (6) is largely based on the ~326 series nano-bridge multi-heterojunctions between source and drain junctions, across the channel with 10 µm width. Each nanoscale mono-/multi-layer heterojunction appears to act as a pseudo-one-dimensional energy bridge between the source and drain junctions by the built-in electric field, interlayer gap, and work function difference, by which electrons and holes are effectively separated and drifted along the nanowire-like energy bridges. Therefore, the improved photoresponsivity have been obtained by maintaining the high mobility in the channel structure in Type (6) [serial nano-bridge multi-heterojunction] and reducing the carrier transit time to be shorter than those in Type (3)–(5). As shown in Fig. 3g, the rise/decay times for Type (5) and Type (6) were extracted to be (12.31/13.705 s) and (0.02/0.025 s), respectively. Therefore, the photoresponse rise/decay times for Type (6) were significantly improved to be 486.5/632.2 times shorter, in comparison with Type (2) multi-layer device, while those for Type (5) having the parallel nano-bridge heterojunctions on the multi-layer MoS_2_ channel were comparable or degraded in comparison with Type (2). For the photoresponse rise/decay times of Type (6), the carriers in the depletion regions (~326) residing in the multiple heterojunctions of the mono-/multi-layer structure are effectively swept by the electric field and make up the drift current, with the fast rise/decay times of photocurrent^46,48,49^, and the charge traps formed on the edges of the serial nano-bridge multi-heterojunction (band-gap of the MoS_2_ surface region) substantially increase the recombination rate of photocarriers, and consequently, reduce the photocarrier lifetime^50^. Therefore, in Type (6), since the carrier transit time (*τ*_transit_) is much shorter than the carrier lifetime (*τ*_life_), a high photoconductive gain (*G*) can be obtained [*τ*_transit_ = *L*^2^/(*μ*_FE_ *V*_d_) = 3.3 ns, where, *L* is the channel length (7.5 µm), *μ*_FE_ is the field-effect mobility (34.54 cm^2^ V^−1^ s^−1^), and *V*_d_ is the applied drain voltage (5 V). *G* = *τ*_life_/*τ*_transit_ = 7.58 × 10^6^, where the value of *τ*_life_ (25 ms) was extracted from the photoresponse curve (decay time) of Type (6)]. (Even though the carrier lifetime is shorter than the decay time due to the carrier trap in the MoS_2_, because the decay time is proportional to the carrier lifetime, the decay time was inserted as the carrier lifetime in the calculation of *G* value, However, due to the presence of charge traps, the carrier lifetime becomes shorter than the decay time of the photodetector^51^, thus, the calculated *G* might be larger than the actual *G*.) Fig. 3h-j are the comparison of photoresponsivities (at *V*_g_ = 0 V) and photoresponse rise time/decay time extracted from Fig. 3b-g for the MoS_2_ FETs of Type (1)–(6). In addition, the hysteresis comparison between Type (2) and Type (6), the output curve and the *I*_d_–*V*_g_ characteristics (as a function of *V*_d_) according to laser on-/off-state of Type (6), and the photoresponsivity of Type (1)–(6) extracted as a function of *V*_g_ can be seen in Supplementary Note 7.

### Comparison of optoelectronic performances measured for the different number of heterojunctions, for the different number of selective ALE cycles, for different incident power, for different wavelength, and with previous results

**Fig. 4:**
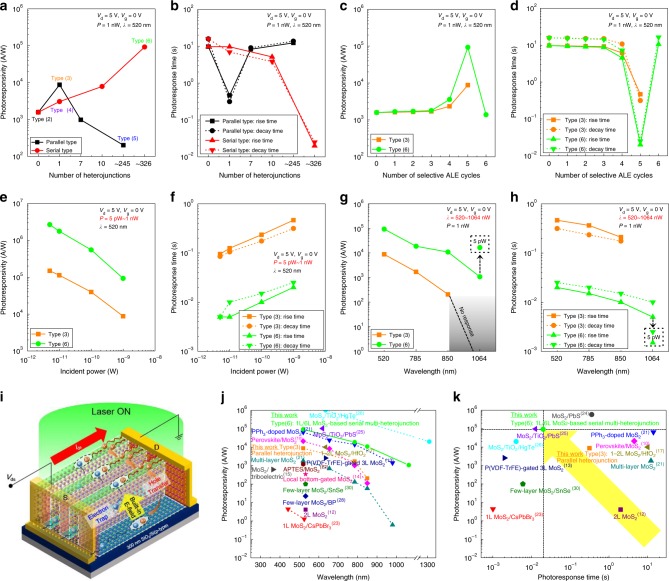
Comparison of optoelectronic performances. **a** Photoresponsivity and **b** photoresponse time according to the number and direction of heterojunctions in the MoS_2_ channel. **c** Photoresponsivity and **d** photoresponse time of Type (3) and (6) according to the number of selective ALE cycles patterned on 6L-MoS_2_. **e** Photoresponsivity and **f** photoresponse time of Type (3) and (6) as a function of the incident laser power. **g** Photoresponsivity and **h** photoresponse time of Type (3) and (6) as a function of the laser wavelength. **i** Schematic drawing on carrier transport mechanism of Type (6) photodetector. Quantitative comparison of **j** photoresponsivity and **k** photoresponse time with previously reported MoS_2_-based photodetectors

The effects of the number of parallel-type and serial-type mono-/multi-layer (6L) MoS_2_ heterojunctions in the 10 µm width MoS_2_ channel on the photoresponsivity and photoresponse time are shown in Fig. 4a, b. The multiple parallel mono-/multi-layer heterojunctions and serial mono-/multi-layer heterojunctions were formed by patterning 1 μm width PR with 2 μm pitch on the 10 µm width MoS_2_ channel followed by selective 5 ALE cycles. The formation of multiple mono-/multi-layer heterojunctions could be identified by Raman mapping (Supplementary Note 8). As shown in Fig. 4a, for the mono-/multi-layer (6L) heterojunctions parallel to the source and drain, the photoresponsivity was increased when the number of heterojunction is increased from 0 [Type (2)] (1.58 × 10^3^ A/W) to 1 [Type (3)] (8.74 × 10^3^ A/W), but the further increase in the parallel mono-/multi-layer heterojunctions to 7 and to ~245 decreased the photoresponsivity gradually to 9.84 × 10^2^ and to 2.02 × 10^2^ A/W, respectively, due to the increased number of energy barriers blocking carrier transport between the source and drain. On the contrary, for the mono-/multi-layer (6L) heterojunctions serially arranged to the source and drain, as the number of heterojunction is increased from 0 to 1, 10, and to ~326, the photoresponsivity was increased continuously to 3.07 × 10^3^, 7.87 × 10^3^, and to 9.26 × 10^4^ A/W, respectively, due to the increased number of energy bridges connecting the source and drain. In the case of photoresponse time, as shown in Fig. 4b, for the parallel mono-/multi-layer heterojunctions, the photoresponse rise time/decay time were the shortest when the number of the parallel heterojunctions was 1 (0.465/0.315 s) but the further increased number of parallel heterojunctions to 7 and to ~245 increased the rise time/decay time to (8.275/9.055 s) and to (12.31/13.705 s), respectively, due to the increased charge scattering by the energy barriers. But, for the serial mono-/multi-layer heterojunctions, as the number of heterojunctions was increased from 0 to 1, 10, and to ~326, the rise time/decay time were gradually decreased to (9.655/6.93 s), (5.135/3.835 s), and to (0.02/0.025 s), respectively, due to the increased number of depletion regions with built-in electric fields in the multiple heterojunctions serially connecting the source and drain as mentioned above. We also fabricated a serial type multi-heterojunction photodetector (mono-/multi-layer structure with 1 μm width and 2 μm pitch) in a mechanically exfoliated multi-layer MoSe_2_ to verify the applicability to other TMD materials and confirmed the similar improvement of the photoresponse characteristics after the formation of multi-heterojunction (Supplementary Note 9).

For the Type (3)-parallel heterojunction and Type (6)-serial nano-bridge multi-heterojunction, the number of ALE cycles was varied from 0 to 6 for the formation of (6~0L) layer/multi-layer (6 L) MoS_2_ heterojunctions in the 10 µm width and 7.5 µm length MoS_2_ channel and the effects of the number of ALE cycles on the photoresponsivity and photoresponse time were investigated and the results are in Fig. 4c, d. The schematic drawings of the different layer (6~1L)/multi-layer (6L) MoS_2_ heterojunctions are presented in Supplementary Note 10. As shown in Fig. 4c, when the ALE cycle was increased from 0 to 3 (that is, for (6~3L)/6L heterojunctions), the photoresponsivity was not significantly varied and remained at ~1.6 × 10^3^ A/W for both Type (3) and Type (6). But, as the ALE cycle was increased to 4 (that is, for 2L/6L heterojunctions), the slight increase of photoresponsivities to 2.33 × 10^3^ and 3.57 × 10^3^ A/W were observed for Type (3) and Type (6), respectively. And, when the ALE cycle was 5 (that is, for 1L/6L heterojunctions), the highest photoresponsivities of 8.74 × 10^3^ and 9.26 × 10^4^ A/W for Type (3) and Type (6), respectively, were observed. However, the further increase of ALE cycle to 6 for Type (6) [for Type (3), the MoS_2_ channel is disconnected after 6 ALE cycles], decreased the photoresponsivity significantly to 1.38 × 10^3^ A/W due to the change to a multi-layer MoS_2_ FET without heterojunctions [similar to Type (2) but ~1/2 channel area]. Therefore, it is found that, the photoresponsivity is the highest when the mono-/multi-layer heterojunction is formed. The photoresponse time in Fig. 4d exhibited similar trend as the photoresponsivity. That is, the photoresponse rise time/decay time were the shortest (that is, the response speed is the fastest) for the MoS_2_ FETs with mono-/multi-layer heterojunctions, and the second shortest for the MoS_2_ FETs with bi-/multi-layer heterojunctions while other MoS_2_ FETs with (6~3L)/6L junctions showed the similar long response time.

Using Type (3)-mono-/multi-layer parallel heterojunction and Type (6)-mono-/multi-layer serial nano-bridge multi-heterojunction, the effects of incident laser power (5 pW–1 nW) and wavelength (520–1064 nm) on the photoresponsivity and photoresponse time were investigated. Figure 4e, f show the photoresponsivity and photoresponse time measured as a function of laser (*λ* = 520 nm) incident power from 5 to 1000 pW (1 nW), respectively, for both Type (3) and Type (6). With the decrease of laser incident power from 1000 to 5 pW, the increased photoresponsivity and decreased photoresponse time were observed for both Type (3) and Type (6) FETs (photodetectors). The improved photoresponse characteristics at the lower laser power density are known to be due to the presence of trap states in MoS_2_ ^7^ and the suppressed scattering of photocarriers^22^ at the lower incident laser power. However, in Fig. 4f, the suppressed scattering of photocarriers seems to be more dominant for the gradual decrease in photoresponse time. Especially, in our study, at the lowest incident power of 5 pW, the most excellent properties of photoresponsivity and photoresponse time (rise time/decay time) were observed for Type (6) [2.67 × 10^6^ A/W and (5 ms/5 ms)].

The effects of laser wavelength at the laser power of 1 nW on the photoresponsivity and photoresponse time are shown in Fig. 4g for the wavelength from 520 to 1064 nm. For Type (3), as the laser wavelength was increased from 520 to 850 nm, the photoresponsivity was gradually decreased from 8.74 × 10^3^ to 2.05 × 10^2^ A/W, and for laser wavelength of 1064 nm, the Type (3) did not show any photoresponsivity. However, in the case of Type (6), even though the photoresponsivity was also decreased with the increase of laser wavelength, still high photoresponsivity of 1.07 × 10^3^ A/W was observed at the wavelength of 1064 nm in addition to high photoresponsivities of 9.26 × 10^4^, 1.86 × 10^4^, and 1.08 × 10^4^ A/W at the wavelength of 520, 785, and 850 nm, respectively. Therefore, Type (6) photodetector exhibited a broadband light detection range (visible–NIR) in addition to high photoresponsivity at the wavelength range. In the case of the photoresponse time for Type (3) and Type (6), as shown in Fig. 4h, it was also decreased with the increase of wavelength for both types and, especially, for Type (6), the rise time/decay time were the shortest [5 ms/10 ms] at 1064 nm because the photocarriers detected at the NIR range are dependent on interlayer gap not on band-to-band transition^52^. In addition, as shown in Fig. 4g, h, when the incident power was lowered to 5 pW at the wavelength of 1064 nm, the photoresponsivity and photoresponse time (rise time/decay time) of Type (6) were further improved to 1.65 × 10^4^ A/W and (1.5 ms/2.5 ms), respectively.

Therefore, it is found that, by using the serial nano-bridge mono-/multi-layer multiple heterojunctions, not only increased photocarrier generation rate but also faster photoresponse (rise/decay times) and carrier transport could be obtained through the construction of pseudo-one-dimensional energy bridges of the mono-/multi-layer heterojunctions with the seriously connected source and drain junctions sustained by the built-in electric field in the depletion region. The performance improvements also attribute to the interlayer gaps including direct gaps, work function difference, and effectively maintained high mobility across the multiple heterojunctions. Figure 4i shows the schematic of the photodetector of Type (6) with the illustration of carrier generation and transport mechanisms. The photodetection properties of MoS_2_-based photodetectors reported until now were summarized in Fig. 4j for photoresponsivity and Fig. 4k for photoresponse time to compare the properties more quantitatively for the laser incident power of 1–6 nW and for various wavelengths. As shown in Fig. 4j, k, the MoS_2_ photodetector with the serial nano-bridge multi-heterojunctions investigated in this study exhibited the widest photoresponse wavelength range and the highest photoresponsivity while maintaining fast photoresponse just by changing the MoS_2_ FET channel structure from one multi-layer structure to the structure with serial multiple mono-/multi-layers. The details of each photodetector in Fig. 4j, k can be found in Supplementary Data 1. In addition, the detectivity for each photodetector was also compared with Type (6) photodetector (Supplementary Data 2).

## Discussion

In summary, we fabricated MoS_2_-based photodectors with serial nano-bridge MoS_2_ multi-heterojunctions consisted of a mono-/multi-layer structure through the selective layer control of multi-layer MoS_2_ FET channel. It was confirmed that the formation of nanowire-like energy bridges between the source and drain (serial nano-bridges) by the built-in electric field in the depletion region, work function differences (*Φ*_mono_: 4.45 eV and *Φ*_multi_: 4.62 eV), interlayer gap between the alternating mono- and multi-layers separated by a nanoscale distance, and maintaining high mobility, not only significantly enhanced photoconductivity but also fast photoresponse could be realized by transporting the electrons and holes separately along the nanowire-like energy bridges. When the effects of the number of layer combination in the heterojunction structure, direction (parallel- and serial-type), and number (1 to ~326) of heterojunctions between the source and drain were investigated, the improved photoresponse characteristics were observed as the heterojunction layer structure was changed from 6L/6L to 1L/6L, as the number of heterojunction between the source and drain was increased from 1 to ~326, and for the serial-type instead of parallel-type. For the MoS_2_ heterojunctions consisted of serial-type, ~326, and mono-/multi-layer (6L) heterojunctions, the best photoresponsivity (2.67 × 10^6^ A/W at *λ* = 520 nm and 1.65 × 10^4^ A/W at *λ* = 1064 nm) and the fast photoresponse (rise/decay times: 5 ms/5 ms at *λ* = 520 nm and 1.5 ms/2.5 ms at *λ* = 1064 nm) in the broad detection range (visible–NIR) could be obtained. This serial nano-bridge multi-heterojunction photodetector structure investigated in this study can be easily applied to various next generation TMD-based optoelectronic devices just by an additional selective layer control process for enhanced optoelectronic properties.

## Methods

### Fabrication of MoS_2_ FETs

For the multi-layer MoS_2_ FET fabrication, mechanically exfoliated MoS_2_ layers were transferred on the 300-nm-thick SiO_2_/heavily boron-doped Si substrate. To more accurately compare the electronic and optoelectronic device characteristics of Type (1)–(6) (Supplementary Note 4), similar size multi-layer (6L) MoS_2_ flakes were selected by Raman spectroscopy and AFM analysis. The source/drain electrode contact areas with a 7.5 μm channel length region were patterned using a photolithographic process. Then, 5-nm-thick Ti and 40-nm-thick Au were deposited sequentially by an e-beam evaporator.

### Characterization of electronic and optoelectronic properties

The current–voltage (*I*_d_–*V*_g_) characteristics for photodetectors fabricated with Type (1)–(6) were measured using the Keysight B2912A semiconductor parameter analyzer in a vacuum probe station. Here, all *I*_d_ were normalized by the channel width. Light sources were dot lasers (Delos Laser) with the wavelengths of 520, 785, 850, and 1064 nm and the optical power (*P*_laser_) of 1.33 mW/cm^2^. The photocurrent (*I*_photo_) and photoresponsivity (*R*) were calculated from *I*_d_–*V*_g_ under both dark and illuminated conditions to measure the optoelectronic performance (*I*_laser_on_ − *I*_laser_off_ = *I*_photo_), (*I*_photo_/*P*_laser_ = *R*). The photoresponse (rise time/decay time) characteristics of the photodetectors of Type (1)–(6) were analyzed in the photoswitching cycle (20 s of laser on-state and off-state), and the photocurrents (*I*_max_) were nomalized to their peak values for easy comparison. The rise time and decay time were extracted from (10 to 90%) and (90 to 10%) of the measured *I*_max_^53^, respectively, and a more detailed description can be found in Supplementary Note 5. The field-effect mobility (*μ*_FE_) was extracted using the following equation: *μ*_FE_ = (∂*I*_d_/∂*V*_g_) × [*L*/(*WC*_ox_*V*_d_)], where, *L* and *W* are the channel length and width (*L* and *W* were obtained from the SEM images of the fabricated MoS_2_ FETs), respectively. *C*_ox_ is the gate oxide capacitance (*C*_ox_ = *ε*_0_*ε*_r_/*d*; *ε*_0_ = 8.85 × 10^−12^ F m^−1^, *ε*_r_ = 3.9, and *d* = 300 nm). Detectivity (D*) was calculated from the D* = (*AB*)^1/2^/NEP, where, *A* is the effective area of the photodetector), *B* is the bandwidth of the photodetector, and NEP is the noise equivalent power. (Supplementary Note 11). We measured the noise spectral density (*S*_n_) of the drain current under dark conditions using a SR570 low-noise current preamplifier^54^.

### Characterization of physical and chemical properties

Raman and PL spectra for the estimation of the number of layers and optical band-gap of MoS_2_ according to the selective layer control were measured using Raman microscopic system (WITEC α 300 M + ) with a wavelength of 532 nm. The surface morphology of MoS_2_ was measured both by AFM (Dimension 3100, Veeco) with a tapping mode and by FE-SEM (S-4700, Hitachi). The atomic structure of the mono-layer MoS_2_ fabricated by the ALE process was observed using a high-angle annular dark-field scanning transmission electron microscopy (HAADF STEM, JEOL JEM ARM 200 F) after transferring the mono-layer MoS_2_ fabricated by the ALE process to the TEM grid. To investigate the chemical composition and oxidation of MoS_2_ surfaces before and after the selective layer control, MoS_2_ surfaces were analyzed using XPS (MultiLab 2000, Thermo VG, Mg K source) with a small area (SA)-XPS mode, and the peak energies were calibrated by the C 1 s peak at 284.5 eV. KPFM analysis using Cr/Au coated Si tip was performed to measure the work function and to estimate the depletion region in the heterojunction of a mono-/multi-layer structure. First, the work function of the KPFM tip (*Φ*_tip_) was calibrated by measuring the contact potential difference between the KPFM tip and the HOPG surface (*Φ*_tip_ − *Φ*_HOPG_ = Δ*V*_CPD_HOPG_) because the work function of HOPG is well known (*Φ*_HOPG_ = 4.6 eV). Then, the work functions of the mono- and multi-layer MoS_2_ surfaces have been calculated by measuring the difference between contact potentials of KPFM tip and MoS_2_ surface (*Φ*_tip_ − *Φ*${}_{{\mathrm{MOS}}_{2}}$ = Δ*V*${}_{{\mathrm{CPD}}\_{\mathrm{MOS}}_{2}}$).

## Supplementary information

Supplementary Information

Description of Additional Supplementary Files

Supplementary Data 1

Supplementary Data 2

## Data Availability

The data that support the findings of this study are available from the corresponding author upon reasonable request.
