# Supplementary material for: Ultrasensitive MoS2 photodetector by serial nano-bridge multi-heterojunction
Source: Nat Commun. 2019 Oct 16;10:4701. doi: 10.1038/s41467-019-12592-w (PMC6796006; doi:10.1038/s41467-019-12592-w)
Supplement: Supplementary file 1 — Supplementary Information [file 41467_2019_12592_MOESM1_ESM.pdf]

Supplementary Information

Ultrasensitive MoS_2 photodetector by serial nano-bridge multi-heterojunction

Ki Seok Kim [1], You Jin Ji [1], Ki Hyun Kim [1], Seunghyuk Choi [2], Dong-Ho Kang [3,4], Keun Heo [4], Seongjae Cho [5], Soonmin Yim [6,7], Sungjoo Lee [2], Jin-Hong Park [4], Yeon Sik Jung [7], and Geun Young Yeom [1,2,]*

[1.] *School of Advanced Materials Science and Engineering, Sungkyunkwan University, 2066 Seobu-ro, Jangan-gu, Suwon-si, Gyeonggi-do 16419, Republic of Korea.*
[2.] *SKKU Advanced Institute of Nano Technology (SAINT), Sungkyunkwan University, 2066 Seobu-ro, Jangan-gu, Suwon-si, Gyeonggi-do 16419, Republic of Korea.*
[3.] *School of Electrical and Electronic Engineering, Nanyang Technological University, 50 Nanyang Avenue, 639798 Singapore, Singapore*
[4.] *School of Electronic and Electrical Engineering Sungkyunkwan University, 2066 Seobu-ro, Jangan-gu, Suwon-si, Gyeonggi-do 16419, Republic of Korea.*
[5.] *Department of Electronics Engineering, Gachon University, Gyeonggi-do 13120, Republic of Korea.*
[6.] *Pritzker School of Molecular Engineering, The University of Chicago, 5640 South Ellis Avenue Chicago, IL, 60637, United States.*
[7.] *School of Materials Science and Engineering, Korea Advanced Institute of Science and Technology (KAIST), 291 Daehak-ro, Yuseong-gu, Daejeon, 305-701, Republic of Korea.*

*Corresponding Author : gyyeom@skku.edu

Supplementary Note 1: Schematic diagram of atomic layer etching (ALE) process

The ALE process used for the selective layer control can remove one mono-layer MoS_2 by one etch cycle as shown in Supplementary Figure 1a,b (1st step: Cl radical adsorption and 2nd step: Ar^+-ion beam desorption). Cl radicals were chemisorbed on the top-layer MoS_2 to lower the binding energy between top Cl-adsorbed MoS_2 layer and the rest of MoS_2 layers, and only the top Cl-adsorbed MoS_2 layer with a relatively lower binding energy was easily removed with a controlled Ar^+-ion beam without further removing the remaining MoS_2 layers. Thus, one mono-layer MoS_2 was removed per each ALE cycle.

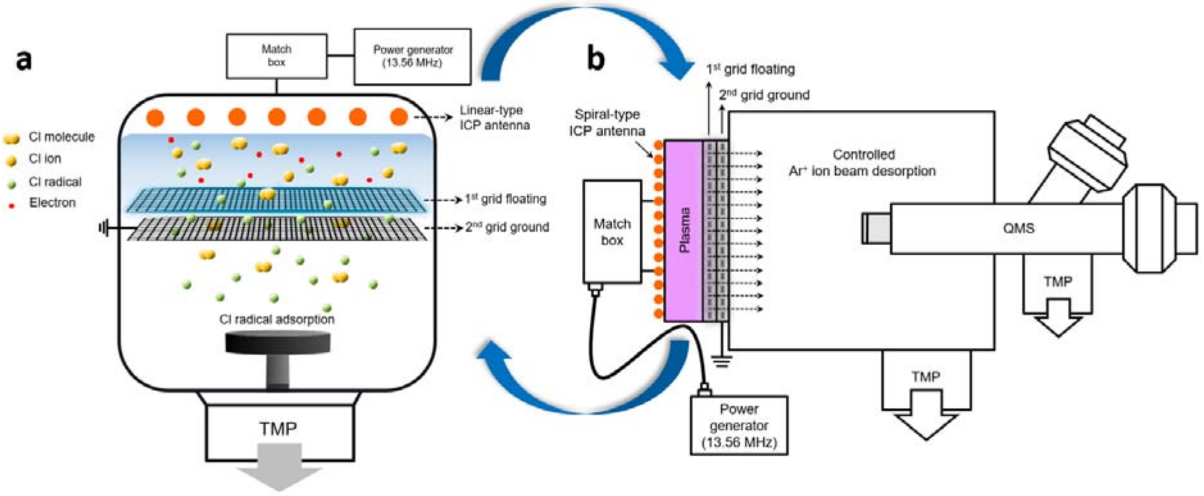

Supplementary Figure 1. Schematic diagram of the ALE process for selective layer control. **a, 1st step:** Cl radical adsorption by an chlorine inductively coupled plasma (ICP) with metal mesh grids. **b, 2nd step:** Removal of Cl-adsorbed MoS_2 one mono-layer by using a two-grid Ar^+-ion beam.

Supplementary Note 2: X-ray photoelectron spectroscopic (XPS) analysis of MoS_2 surfaces

Supplementary Figure 2a shows no change in the XPS data of Mo 3d and S 2p between the pristine 6L-MoS_2 and the 6L-MoS_2 after 5th cycle ALE. However, Mo^{6+} was observed at ~236 eV after an O_2 plasma exposure (120 s at 6.7 mTorr and 70 sccm of O_2 and 200 W of a 13.56 MHz ICP power) on the pristine 6L-MoS_2 surface. The Mo^{6+} peak appears when the MoS_2 surface is oxidized and indicates the changes of the MoS_2 intrinsic properties[1,2]. Supplementary Figure 2b shows XPS data of Cl 2*p* when one cycle ALE process is performed on the pristine 6L-MoS_2. Cl peaks ($2p_{1/2}$ and $2p_{3/2}$) were completely removed after Ar^+-ion beam desorption (2nd step) was performed on Cl-adsorbed MoS_2, indicating no etch residue on the MoS_2 surface after the ALE process. Supplementary Figure 2c shows the comparison of the S/Mo ratio. The S/Mo ratios of pristine 6L-MoS_2, after 5th cycle ALE and after the O_2 plasma exposure were 2.023, 2.012 and 1.547, respectively, indicating that the S/Mo ratio of the MoS_2 layer is preserved even after the 5th cycle ALE while the ratio is changed after the oxygen plasma exposure. Supplementary Figure 2d shows the change of the XPS binding energy positions of the Mo peaks ($3d_{3/2}$ and $3d_{5/2}$) and S peaks ($2p_{1/2}$ and $2p_{3/2}$). The XPS binding energy positions of pristine 6L-MoS_2 and that after 5th cycle ALE were all the same, but after the O_2 plasma exposure, both Mo and S peaks were red-shifted. In general, the red shift is observed when the MoS_2 surface is oxidized and damaged[2].

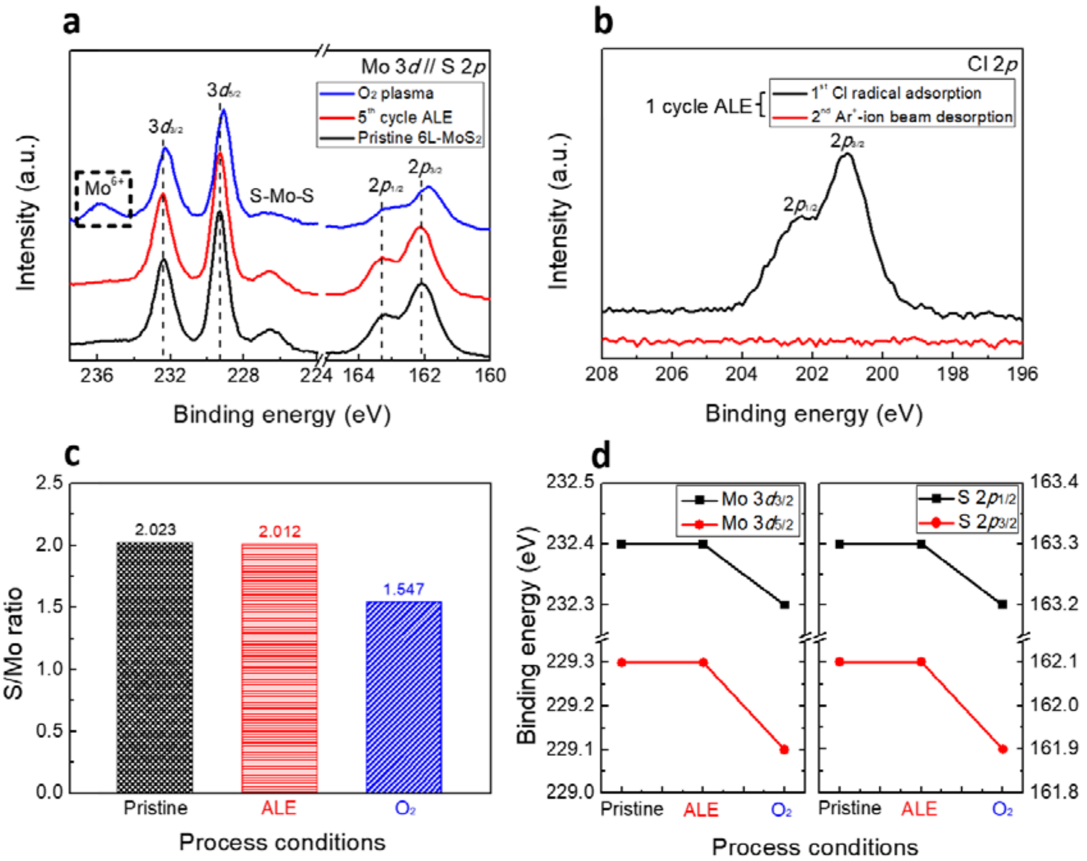

Supplementary Figure 2. XPS analysis of MoS_2 surface characteristics before/after the MoS_2 ALE or an O_2 plasma exposure. **a,** XPS data of Mo 3*d* and S 2*p* for pristine 6L-MoS_2, after 5th cycle ALE, and after an O_2 plasma exposure. **b,** XPS data of Cl 2*p* after Cl radical adsorption and after Ar^+-ion desorption. **c,** Change of the S/Mo ratio for pristine 6L-MoS_2, after 5th cycle ALE, and after an O_2 plasma exposure. **d,** Change of the XPS binding energy positions of the Mo peaks ($3d_{3/2}$ and $3d_{5/2}$) and S peaks ($2p_{1/2}$ and $2p_{3/2}$) for pristine 6L-MoS_2, after 5th cycle ALE, and after O_2 plasma exposure. O_2 plasma was generated for 120 s at 6.7 mTorr/70 sccm of O_2 and 200 W of a 13.56 MHz ICP power.

Supplementary Note 3: Properties of MoS_2 layers prepared by different methods

In case of the pristine 6L-MoS_2, an indirect bandgap of 1.394 eV was obtained by PL measurement at room temperature as shown in Supplementary Figure 3a (The intensity of 6L-MoS_2 in the indirect gap is 5 times magnified). On the other hand, the indirect bandgap is not observed from the 1L-MoS_2 formed after the 5th-cycle ALE process since a local minimum in the conduction band which prepares an indirect bandgap is not effectively constructed[3]. Supplementary Figure 3b–d demonstrate PL spectra and I_d-V_g curves of the devices based on mono-layer MoS_2 and 6L-MoS_2 after 5 ALE cycles, where almost identical characteristics are shown within a small error range. The formation of the mono-/multi-layer heterojunction with the staggered band alignment having an interlayer gap (narrow gap) between 1L- and 6L-MoS_2 layers has been explicitly identified. As shown in Supplementary Figure 3e, the STEM image of the mono-layer MoS_2 fabricated by the ALE process reveals that Mo and S atoms are uniformly arranged in a high periodicity to form a trigonal prismatic (2H)[4]. Supplementary Figure 3f shows an enlarged image of the area in the red box in Supplementary Figure 3e and the Mo and S intensity profiles are visualized along the green line.

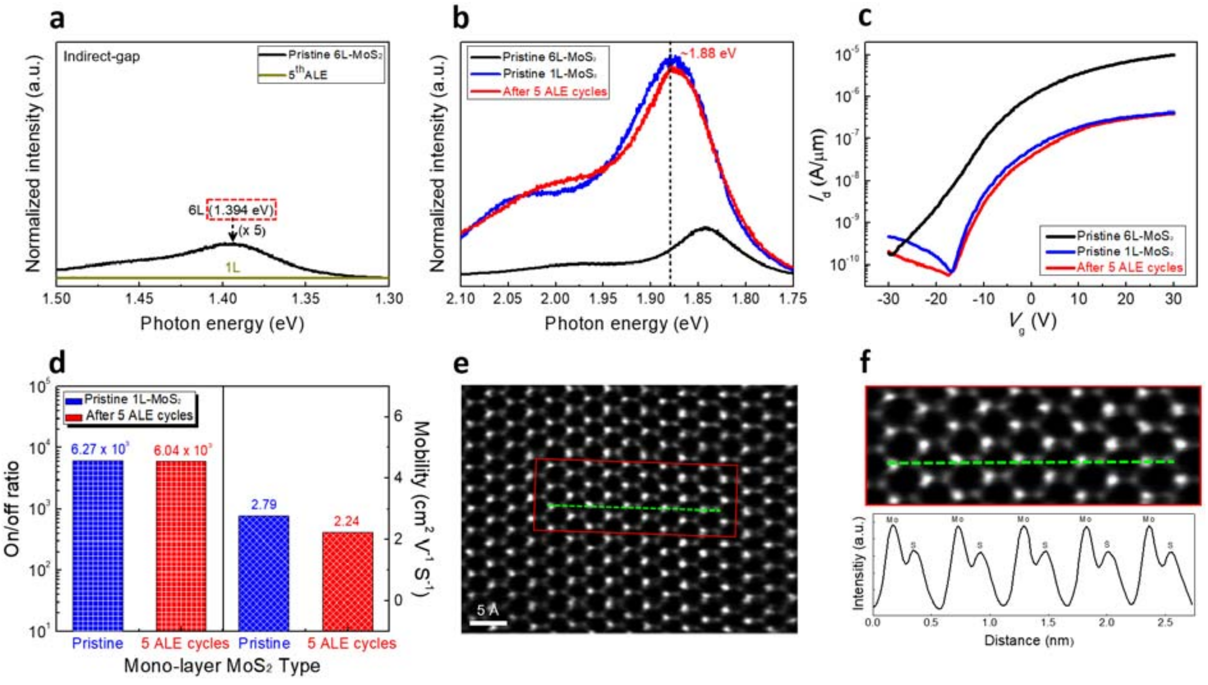

Supplementary Figure 3. Comparison of the properties between pristine mono-layer MoS_2 and 6L-MoS_2 after 5 ALE cycles. **a,** PL spectra analyses of 1L- and 6L-MoS_2. Comparison between **b,** PL spectra and **c,** I_d–V_g curves obtained from the pristine 6L-MoS_2, the 6L-MoS_2 after 5 ALE cycles, and the pristine mono-layer MoS_2. **d,** Electrical characteristics of the 6L-MoS_2 after 5 ALE cycles and the pristine mono-layer MoS_2. **e,** STEM image of the mono-layer MoS_2 fabricated by the ALE process. **f,** Enlarged image of the region in the red box in **e** and the intensity profile along the green line.

Supplementary Note 4: Fabrication processes for the MoS_2-based FETs of Type (1)~(6)

In this study, similar size (~10 μm by ~10 μm) mono- and multi-layer MoS_2 were used to clearly compare the electronic and optoelectronic device chracteristics of Type (1)~(6) as shown in Supplementary Figure 4. Also, 6-layer (6L) MoS_2 was selected as the multi-layer MoS_2. Before the selective ALE process, PR half patterning parallel and vertical to the source/drain edge was performed using an photolithography for a parallel mono-/multi-layer heterojunction [Type (3)] and a serial mono-/multi-layer heterojunction [Type (4)], respectively. SiO_2 line patterns was formed using a nano-pattering process [solvent-assisted nanotransfer printing (S-nTP) process][5] parallel and vertical to the source/drain edges for two mono-/multi-layer MoS_2 multiple heterojunctions (parallel nano-bridge [Type (5)] and serial nano-bridge [Type (6)]. Through the nano-patterning process, the SiO_2 line pattern with the line width of 25 nm could be formed at 50 nm intervals. ALE process was used to form heterojunctions of mono-/multi-layer structure through the selective layer control, and the PR and SiO_2 patterns were cleanly removed using aceton and a HF (HF:deionized water = 1:100, etch time = 10 s) solution, respectively.

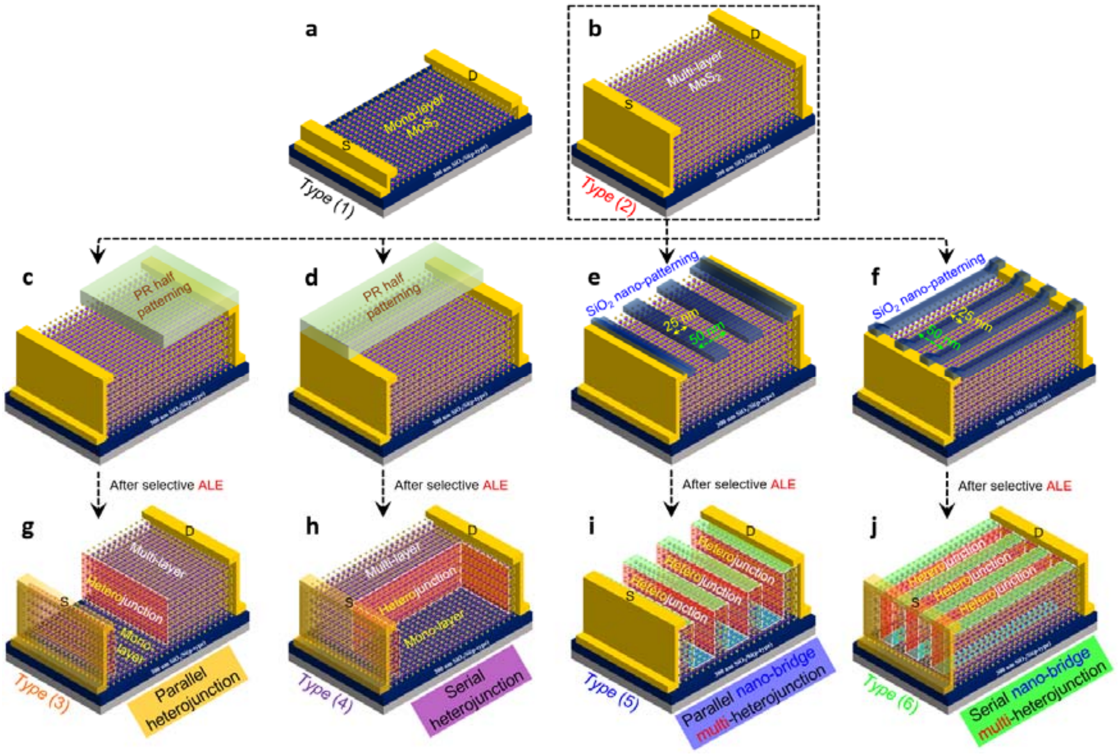

Supplementary Figure 4. Schematic diagram for the MoS_2-based FETs of Type (1)~(6). **a,** Pristine mono-layer MoS_2 FET [Type (1)] and **b,** Pristine multi-layer MoS_2 FET [Type (2)]. **c,** Parallel- and **d,** serial-type PR half patterning using a photolithography on multi-layer MoS_2 FETs. **e,** Parallel- and **f,** serial-type SiO_2 nano-bride patterning using a nano-patterning process on multi-layer MoS_2 FETs. MoS_2 back-gated FETs (photodetectors) of **g,** Type (3)-parallel, **h,** Type (4)-serial, **i,** Type (5)-parallel nano-bridges, and **j,** Type (6)-serial nano-bridges fabricated with the selective layer control using ALE process.

Supplementary Note 5: Photoresponse time extraction

The photoresponse time (rise and decay) characteristics of the photodetector were analyzed in a laser on/off cycle (20 s of laser on-state and 20 s of laser off-state), and the maximum photocurrents (I_{max}) were normalized as 1.0 for the accurate comparison of Type (1)~(6). The rise time and decay time were extracted from (the time between $I_{10\%}$ to $I_{90\%}$) and (the time between $I_{90\%}$ to $I_{10\%}$) of the measured I_{max}. Therefore, for Type (1), the rise time and decay time were calculated to be 13.635 s (Supplementary Figure 5a) and 6.84 s (Supplementary Figure 5b), respectively.

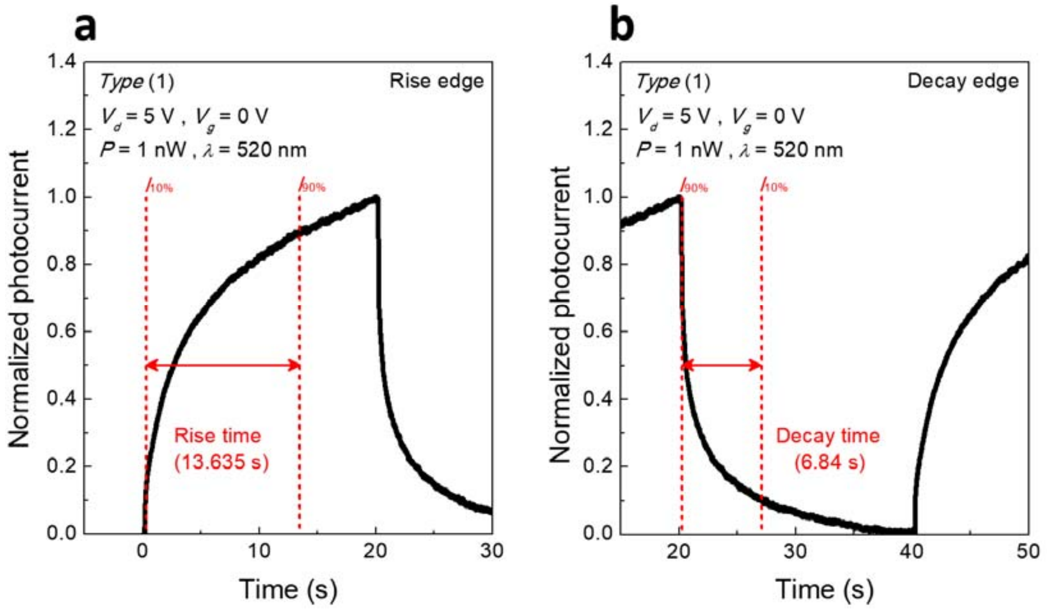

Supplementary Figure 5. Method of photoresponse time (rise and decay) extraction. **a,** Rise and **b,** decay edges of the photoresponse curve obtained for Type (1)-mono-layer MoS_2 FET (photodetector).

Supplementary Note 6: Electrical characterization

Here, the electrical properties of Type (5)-parallel nano-bridge multi-heterojunction were remarkably degraded and were similar to Type (1)-mono-layer possibly due to the series of energy barriers between the source and drain which block the carrier transport. On the other hand, in Type (6)-serial nano-bridge multi-heterojunction, it is possible to maximize the optoelectronic performances without changing electrical characteristics by showing similar electrical characteristics as Type (2)-multi-layer due to no energy barrier such as Type (5) for the carrier transport between the source and drain (Supplementary Figure 6).

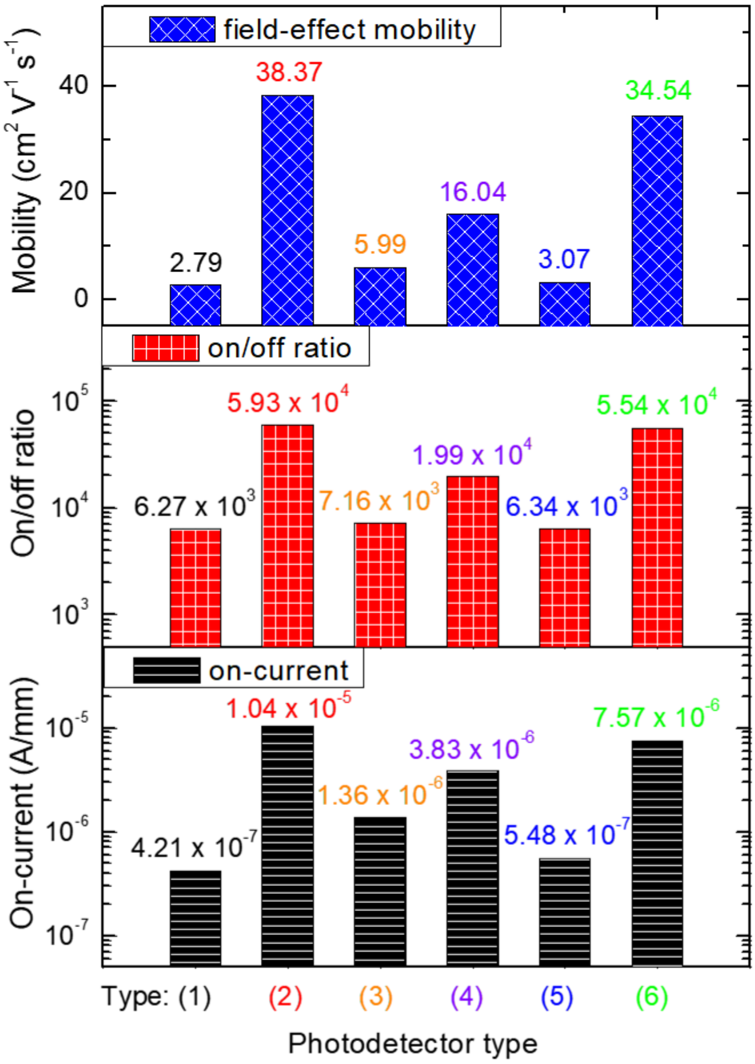

Supplementary Figure 6. The electrical characteristics for the FETs of Type (1)~(6) at the laser-off state. Analysis of on-current, on/off ratio, and field-effect mobility charateristics for the FETs (photodetectors) of Type (1)~(6) (V_d = 5 V, V_g = -30 ~ +30 V and at the laser-off state).

Supplementary Note 7: Comparison of electronic and optoelectronic properties

Supplementary Figure 7a,b show a narrow hysteresis in both Type (2) and Type (6) with a double sweep measured in a vacuum probe station. Supplementary Figure 7c,d show the output curve (V_d = 0 ~ 5 V, V_g = 0 ~ 30 V) of Type (6) for laser on-/off-state. In laser off-state (Supplementary Figure 7c) and laser on-state (Supplementary Figure 7d), the carrier transit time was decreased with increasing V_d, and the effective barrier height was decreased with increasing V_g. Therefore, a gradual increase in I_d is confirmed and acceptable contact quality is also expected[6]. In addition, the reason why the change of I_d as function of V_g is smaller in laser on-state (Supplementary Figure 7d) than laser off-state (Supplementary Figure 7c) is because the strong built-in electric field in the serial nano-bridge multi-heterojunction [Type (6)] generates a large amount of photocarriers even at a small gate voltage. This tendency is also shown in Supplementary Figure 7e–g and previous studies[7–9]. Supplementary Figure 7e–g show the I_d–V_g characteristics of Type (6) as a function of V_d (0.1, 2, and 5 V), and photoresponsivities extracted for V_d = 0.1, 2, and 5 V at V_g = 0 V are 1.14 × 10^3, 1.56 × 10^4, and 9.26 × 10^4 (A/W), respectively. Supplementary Figure 7h shows the photoresponsivity of Type (1)~(6) extracted from Manuscript Figure 3b–d as a function of V_g (V_g = -30 ~ +30 V, V_d = 5 V).

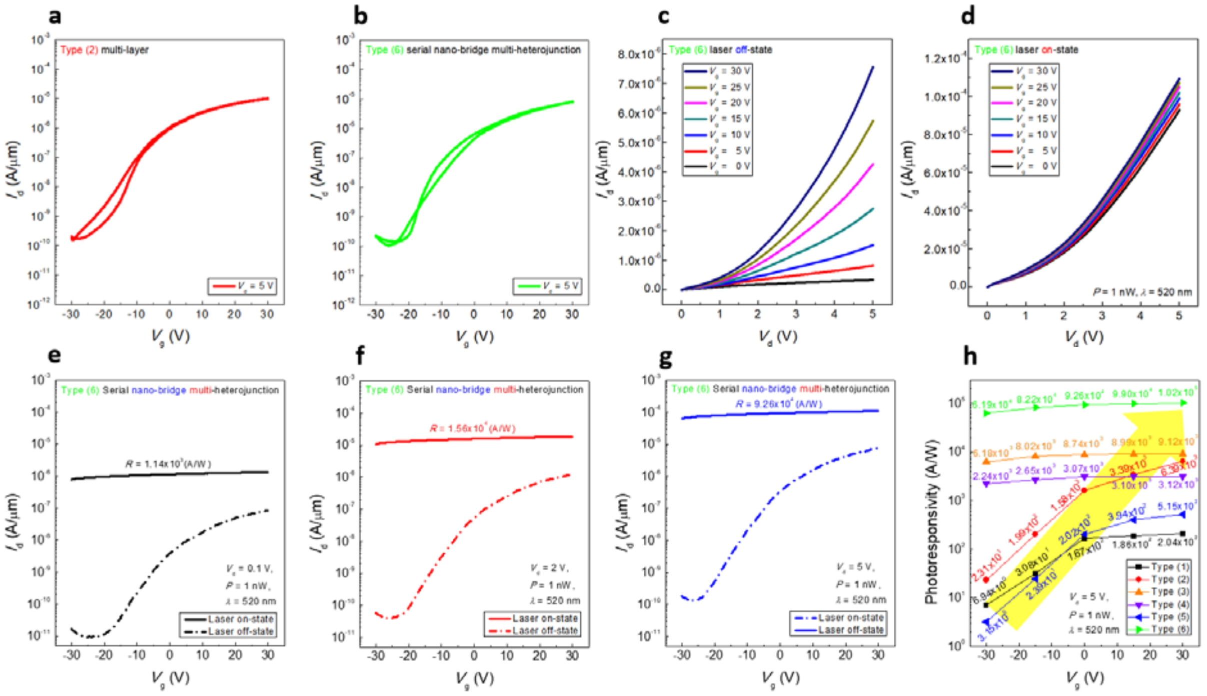

Supplementary Figure 7. Characteristics of hysteresis, output curve (for laser on-/off-state), I_d–V_g (as function of V_d), and photoresponsivity (as function of V_g). The hysteresis comparison between **a,** Type (2) and **b,** Type (6). The output curve of Type (6) according to laser **c,** off-state and **d,** on-state. I_d–V_g characteristics of Type (6) as function of V_d **e,** (0.1 V), **f,** (2 V), and **g,** (5 V). **h,** Photoresponsivity of Type (1)~(6) extracted as a function of V_g (-30 ~ +30 V).

Supplementary Note 8: Optical and Raman mapping images

In order to observe the optoelectronic performance according to the number of parallel-type and serial-type mono-/multi-layer (6L) MoS_2 heterojunctions, 6L-MoS_2 channel was patterned with PR (width of 1 μm) at 2 μm intervals through a photolithographic process and 5 MoS_2 layers were selectively removed by 5 ALE cycles. Optical and Raman mapping images showed that the multi-heterojunctions with the mono-/multi-layer MoS_2 structure were uniformly formed on the ~10 μm width MoS_2 channel (Supplementary Figure 8).

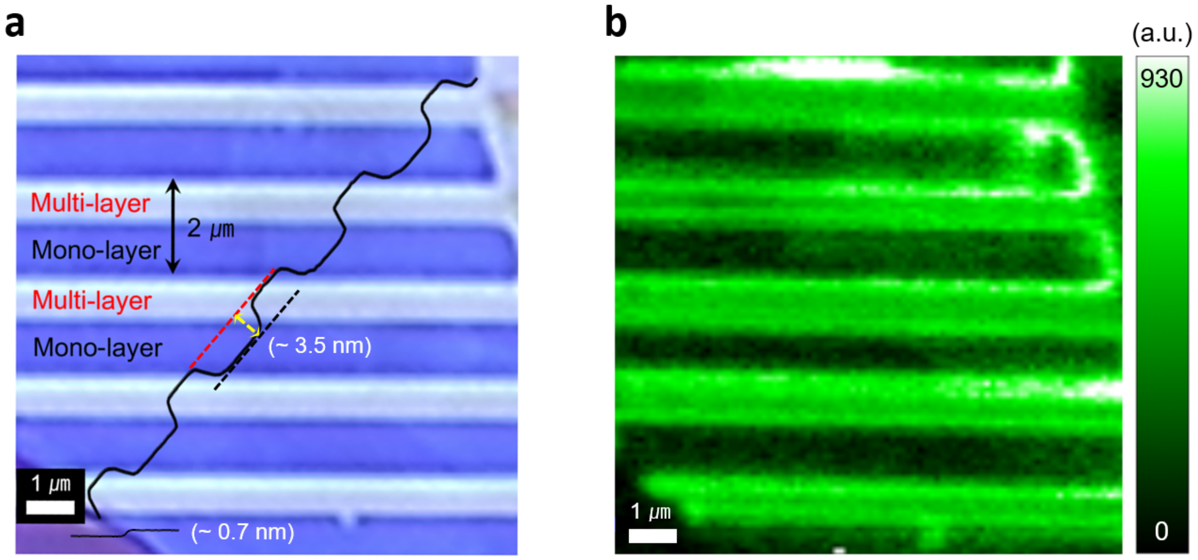

Supplementary Figure 8. Optical and Raman mapping images of multiple heterojunctions fabricated by PR patterning with 1 μm width/ 2 μm pitch followed by 5 ALE cycles. **a,** Optical and **b,** Raman mapping images for a multi-heterojunction of mono-/multi-layer structure.

Supplementary Note 9: Characteristics of $MoSe_2$ photodetector

$MoSe_2$-based FET were fabricated to verify their applicability to other TMD materials. Supplementary Figure 9a shows optical image of a serial type multi-heterojunction fabricated by selective layer control (ALE process) after PR patterning (1 μm width and 2 μm pitch) on a multi-layer $MoSe_2$. Supplementary Figure 9b shows Raman spectra for the black and red dots in Supplementary Figure 9a. It was confirmed that a mono-layer was formed in the PR open region, which is similar to the results of previous studies[10,11]. Supplementary Figure 9c shows the photoresponse curve analysis of $MoSe_2$-based FET before and after selective layer control. When the serial type multi-heterojunction is fabricated, it is confirmed that the photoresponse characteristics are improved due to the enhanced built-in electric field and the reduced charge traps. Here, photoresponsivity (R) and photoresponse time (τ) of the pristine multi-layer $MoSe_2$ and serial multi-heterojunction fabricated by selective layer control are ($R_{pristine} = 2.47 \times 10^3$ A/W and $R_{serial} = 6.95 \times 10^3$ A/W) and ($\tau_{pristine\_rise}/\tau_{pristine\_decay}$ = 10.43 s/>20 s and $\tau_{serial\_rise}/\tau_{serial\_decay}$ = 0.255 s/0.511 s), respectively.

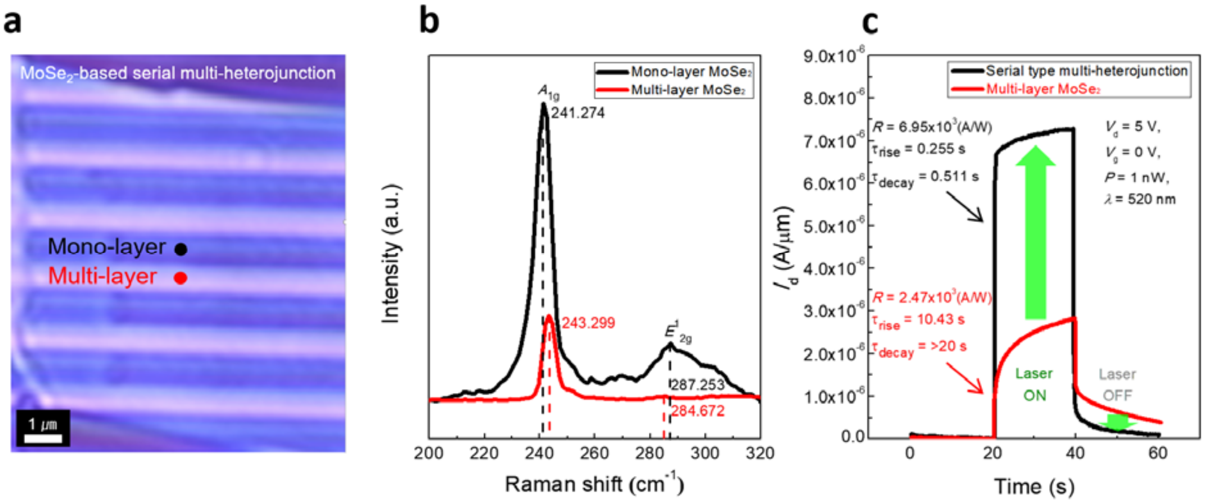

Supplementary Figure 9. Characterization of $MoSe_2$-based serial multi-heterojunction photodetector (mono-/multi-layer structure with 1 μm width and 2 μm pitch). **a,** Optical image for a multi-heterojunction of mono-/multi-layer structure based on $MoSe_2$. **b,** Raman spectra analysis in mono-layer and multi-layer $MoSe_2$. **c,** Comparison of photoresponse curves between pristine multi-layer $MoSe_2$ and serial type multi-heterojunction $MoSe_2$ fabricated by ALE process.

Supplementary Note 10: Schematic diagrams of (6~1L)/(6L) MoS_2 heterojunctions

(6~1L) layer/multi-layer (6L) MoS_2 heterojunctions with the structures of Type (3)-parallel heterojunction and Type (6)-serial nano-bridge multi-heterojunction were formed by selectively removing 6L-MoS_2 patterned with a PR half patterning and SiO_2 line nano-patterning for different numbers of ALE cycles from 0 to 5 (Supplementary Figure 10).

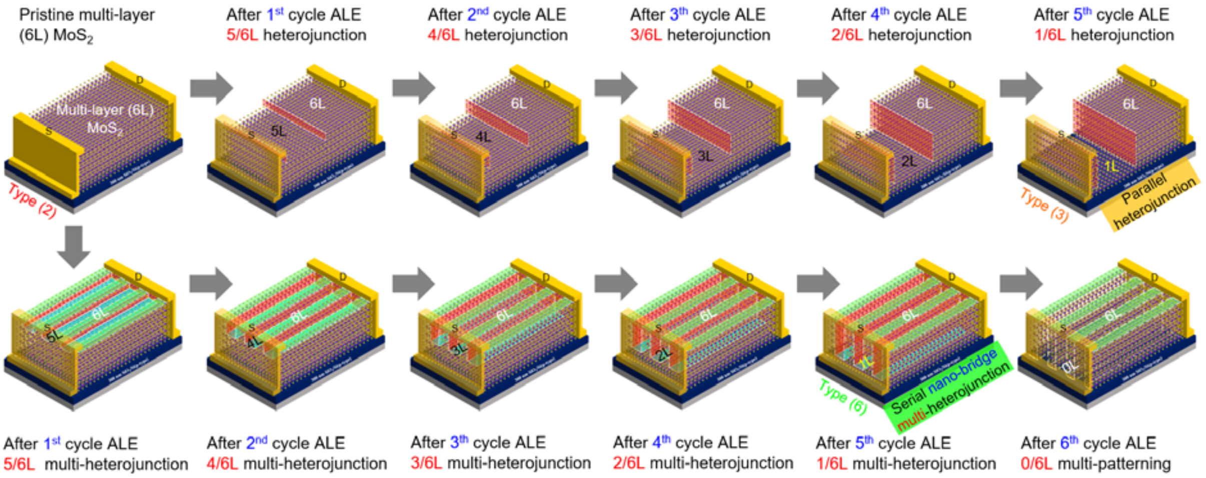

Supplementary Figure 10. Schematic diagrams of (6~1L) layer/multi-layer (6L) MoS_2 heterojunctions by varying the number of selective ALE cycles from 0 to 5. Device structures with increasing selective ALE cycles for Type (3)-parallel heterojunction and Type (6)-serial nano-bridge multi-heterojunction.

Supplementary Note 11: Noise power density of Type (6) photodetector

In order to calculate the detectivity (D^*), we measured the noise power density of the Type (6) serial nano-bridge multi-heterojunction photodetector (Supplementary Figure 11). The noise power density values of the Type (6) photodetector was extracted at 1 Hz. Detectivity (D^*) was calculated from the $D^* = (AB)^{1/2}/NEP = R(A)^{1/2}/S_n$, where, R is the photoresponsivity, A is the effective area of the photodetector, and S_n is the noise spectral density. The measured D^* were 1.93×10^8 jones at $V_g = 0$ V and 2.38×10^{13} jones at $V_g = -25$ V and these were compared with previously reported photodetectors (Supplementary Data 2).

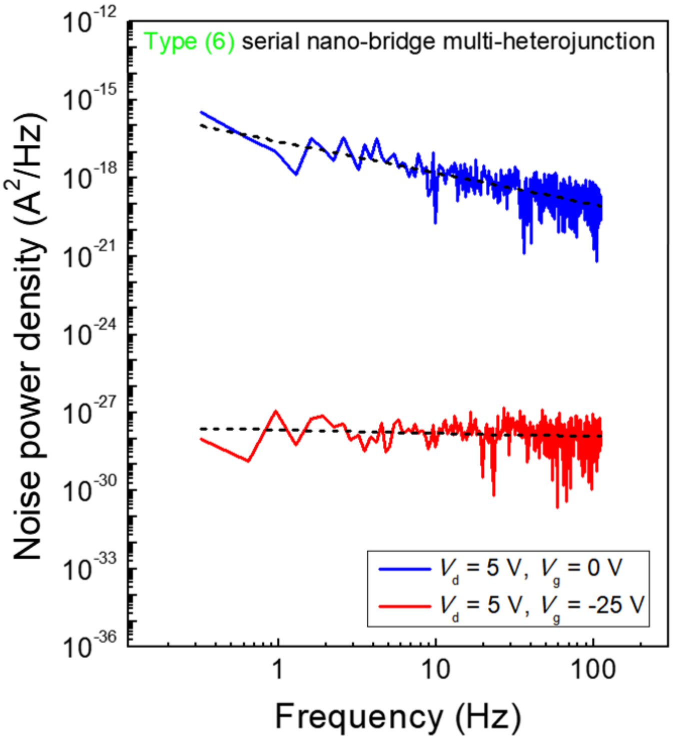

Supplementary Figure 11. Noise spectra of Type (6) serial nano-bridge multi-heterojunction photodetector. Here, the data was measured at $V_d = 5$ V, $V_g = 0$ and -25 V, and fitted 1/f at two gate voltages.

Supplementary References

1. Yang, X. et al. Engineering crystalline structures of two-dimensional MoS_2 sheets for high-performance organic solar cells. *J. Mater. Chem. A* **2**, 7727–7733 (2014).
2. Kim, K. S. et al. Atomic layer etching mechanism of MoS_2 for nanodevices. *ACS Appl. Mater. Interfaces* **9** 11967–11976 (2017).
3. Mak, K. F., Lee, C., Hone, J., Shan, J. & Heinz, T. F. Atomically thin MoS_2: a new direct-gap semiconductor. *Phys. Rev. Lett.* **105**, 136805 (2010).
4. Eda, G. et al. Coherent atomic and electronic heterostructures of single-layer MoS_2. *ACS Nano* **6**, 7311–7317 (2012).
5. Jeong, J. W. et al. High-resolution nanotransfer printing applicable to diverse surfaces via interface-targeted adhesion switching. *Nat. Commun.* **5**, 5387 (2014).
6. Howell, S. L. et al. Investigation of band-offsets at monolayer–multilayer MoS_2 junctions by scanning photocurrent microscopy. *Nano Lett.* **15**, 2278–2284 (2015).
7. Tosun, M. et al. MoS_2 heterojunctions by thickness modulation. *Sci. Rep.* **5**, 10990 (2015).
8. Yang, Y., Huo, N. & Li, J. Sensitized monolayer MoS_2 phototransistors with ultrahigh responsivity. *J. Mater. Chem. C* **5**, 11614–11619 (2017).
9. Pak, J. et al. Improved photoswitching response times of MoS_2 field-effect transistors by stacking p-type copper phthalocyanine layer. *Appl. Phys. Lett.* **109**, 183502 (2016).
10. Tongay, S. et al. Thermally driven crossover from indirect toward direct bandgap in 2D semiconductors: $MoSe_2$ versus MoS_2. *Nano Lett.* **12**, 5576–5580 (2012).
11. Xia, J. et al. CVD synthesis of large-area, highly crystalline $MoSe_2$ atomic layers on diverse substrates and application to photodetectors. *Nanoscale* **6**, 8949–8955 (2014).
12. Wang, W. et al. Hot electron-based near-infrared photodetection using bilayer MoS_2. *Nano Lett.* **15**, 7440–7444 (2015).
13. Furchi, M. M., Polyushkin, D. K., Pospischil, A. & Mueller, T. Mechanisms of photoconductivity in atomically thin MoS_2. *Nano Lett.* **14**, 6165–6170 (2014).
14. Wang, X. et al. Ultrasensitive and broadband MoS_2 photodetector driven by ferroelectrics. *Adv. Mater.* **27**, 6575–6581 (2015).
15. Kwon, J. et al. Giant photoamplification in indirect-bandgap multilayer MoS_2 phototransistors with local bottom-gate structures. *Adv. Mater.* **27**, 2224–2230 (2015).
16. Pang, Y. et al. Tribotronic enhanced photoresponsivity of a MoS_2 phototransistor. *Adv. Sci.* **3**, 1500419 (2016).
17. Zhang, W. et al. High-gain phototransistors based on a CVD MoS_2 monolayer. *Adv. Mater.* **25**, 3456–3461 (2013).
18. Eginligil, M. et al. Dichroic spin–valley photocurrent in monolayer molybdenum disulphide. *Nat. Commun.* **6**, 7636 (2015).
19. Lu, J. et al. Improved photoelectrical properties of MoS_2 films after laser micromachining. *ACS*

Nano **8**, 6334–6343 (2014).

20. Li, B. et al. Direct vapor phase growth and optoelectronic application of large band offset SnS_2/MoS_2 vertical bilayer heterostructures with high lattice mismatch. *Adv. Electron. Mater.* **2**, 1600298 (2016).

21. Wang, B. et al. Enhanced current rectification and self-powered photoresponse in multilayer p-$MoTe_2$/n-MoS_2 van der Waals heterojunctions. *Nanoscale* **9**, 10733–10740 (2017).

22. Ye, L., Li, H., Chen, Z. & Xu, J. Near-infrared photodetector based on MoS_2/black phosphorus heterojunction. *ACS Photonics* **3**, 692–699 (2016).

23. De Fazio, D. et al. High responsivity, large-area graphene/MoS_2 flexible photodetectors. *ACS Nano* **10**, 8252–8262 (2016).

24. Yang, S. et al. Enhanced electrical and optoelectronic characteristics of few-layer type-II $SnSe/MoS_2$ van der Waals heterojunctions. *ACS Appl. Mater. Interfaces* **9**, 42149–42155 (2017).

25. Yang, S. et al. Self-driven photodetector and ambipolar transistor in atomically thin GaTe-MoS_2 p–n vdW heterostructure. *ACS Appl. Mater. Interfaces* **8**, 2533–2539 (2016).

26. Zhou, N. et al. P-GaSe/N-MoS_2 vertical heterostructures synthesized by van der Waals epitaxy for photoresponse modulation. *Small* **14**, 1702731 (2018).

27. Song, X. et al. Boosting two-dimensional $MoS_2/CsPbBr_3$ photodetectors via enhanced light absorbance and interfacial carrier separation. *ACS Appl. Mater. Interfaces* **10**, 2801–2809 (2018).

28. Wang, Y. et al. Solution-processed MoS_2/organolead trihalide perovskite photodetectors. *Adv. Mater.* **29**, 1603995 (2017).

29. Kang, D. H. et al. An ultrahigh-performance photodetector based on a perovskite-transition-metal-dichalcogenide hybrid structure. *Adv. Mater.* **28**, 7799–7806 (2016).

30. Huo, N. & Konstantatos, G. Ultrasensitive all-2D MoS_2 phototransistors enabled by an out-of-plane MoS_2 PN homojunction. *Nat. Commun.* **8**, 572 (2017).

31. Jo, S. H. et al. Broad detection range rhenium diselenide photodetector enhanced by (3-Aminopropyl) triethoxysilane and triphenylphosphine treatment. *Adv. Mater.* **28**, 6711–6718 (2016).

32. Kang, D. H. et al. High-performance transition metal dichalcogenide photodetectors enhanced by self-assembled monolayer doping. *Adv. Funct. Mater.* **25**, 4219–4227 (2015).

33. Zhang, K., Zhai, J. & Wang, Z. L. A monolayer MoS_2 pn homogenous photodiode with enhanced photoresponse by piezo-phototronic effect. *2D Mater.* **5**, 035038 (2018).

34. Kufer, D. et al. Hybrid 2D–0D MoS2–PbS quantum dot photodetectors. *Adv. Mater.* **27**, 176–180 (2015).

35. Kufer, D. et al. Interface engineering in hybrid quantum dot–2D phototransistors. *ACS Photonics* **3**, 1324–1330 (2016).

36. Huo, N., Gupta, S. & Konstantatos, G. MoS_2–HgTe quantum dot hybrid photodetectors beyond 2 μm. *Adv. Mater.* **29**, 1606576 (2017).

37. Pak, S. et al. Consecutive junction-induced efficient charge separation mechanisms for high-performance MoS_2/quantum dot phototransistors. *ACS Appl. Mater. Interfaces* **10**, 38264–38271

(2018).

38. Yu, S. H. et al. Dye-sensitized MoS_2 photodetector with enhanced spectral photoresponse. *ACS Nano* **8**, 8285–8291 (2014).

39. Huang, Y. et al. Van der Waals coupled organic molecules with monolayer MoS_2 for fast response photodetectors with gate-tunable responsivity. *ACS Nano* **12**, 4062–4073 (2018).

40. He, J. et al. Low noise and fast photoresponse of few-layered MoS_2 passivated by $MA_3Bi_2Br_9$. *ACS Photonics* **5**, 1877–1884 (2018).

41. Kufer, D. & Konstantatos, G. Highly sensitive, encapsulated MoS_2 photodetector with gate controllable gain and speed. *Nano Lett.* **15**, 7307–7313 (2015).

42. Choi, W. et al. High-detectivity multilayer MoS_2 phototransistors with spectral response from ultraviolet to infrared. *Adv. Mater.* **24**, 5832–5836 (2012).

43. Sun, M. et al. Lateral multilayer/monolayer MoS_2 heterojunction for high performance photodetector applications. *Sci. Rep.* **7**, 4505 (2017).

44. Ma, J. et al. All polymer encapsulated, highly-sensitive MoS_2 phototransistors on flexible PAR substrate. *Appl. Phys. Lett.* **113**, 013102 (2018).

45. Yoo, G. et al. Flexible and wavelength-selective MoS_2 phototransistors with monolithically integrated transmission color filters. *Sci. Rep.* **7**, 40945 (2017).
