# Supplementary material for: Ultrasensitive MoS2 photodetector by serial nano-bridge multi-heterojunction
Source: Nat Commun. 2019 Oct 16;10:4701. doi: 10.1038/s41467-019-12592-w (PMC6796006; doi:10.1038/s41467-019-12592-w)
Supplement: Supplementary file 2 — Description of Additional Supplementary Files [file 41467_2019_12592_MOESM2_ESM.pdf]

Description of Additional Supplementary Files

File Name: Supplementary Data 1

Description: Comparison of optoelectronic performances (responsivity and rise time/decay time) for MoS2-based photodetectors fabricated with various structures.

File Name: Supplementary Data 2

Description: Comparison of detectivity for MoS2-based photodetectors fabricated with various structures.
